# Temporal Interference Stimulation Targeting the Right Amygdala in Adolescents With Depression: A Pilot Feasibility Study

**DOI:** 10.31083/AP50313

**Published:** 2026-07-15

**Authors:** Fang Wang, Minjia Liu, Hengfen Gong, Shupei Zhou, Fan Wu, Qi Zhang, Lulu Zhang, Na Zhu, Simeng Zhang, Tian Liu, Wei Lu, Jingjing Huang

**Affiliations:** ^1^Clinical Research Center for Mental Disorders, Shanghai Pudong New Area Mental Health Center, School of Medicine, Tongji University, 200124 Shanghai, China; ^2^The Key Laboratory of Biomedical Information Engineering of Ministry of Education, Institute of Health and Rehabilitation Science, School of Life Science and Technology, Xi’an Jiaotong University, 710049 Xi’an, Shaanxi, China

**Keywords:** depressive disorder, adolescent, temporal interference, feasibility studies, amygdala

## Abstract

**Background:**

Adolescent depression necessitates the development of novel therapeutic approaches. Temporal interference stimulation (TIS) is a promising noninvasive neuromodulation technique capable of modulating deep-brain circuits; however, its feasibility in adolescent populations has not yet been established. This open-label pilot study evaluated the feasibility, safety, and tolerability of a five-day TIS protocol targeting the right amygdala in adolescents with depression. Clinical outcomes and neuroimaging findings were assessed as exploratory, hypothesis-generating objectives.

**Methods:**

Twelve adolescents with depression received five consecutive daily sessions of TIS (2000 Hz carrier frequency with a 100 Hz difference frequency; 20 min/day) targeting the right amygdala. Feasibility was evaluated on the basis of tolerability and adherence rates. Clinical outcomes were assessed using the 17-item Hamilton Depression Rating Scale (HAMD-17), Montgomery-Åsberg Depression Rating Scale (MADRS), 14-item Hamilton Anxiety Rating Scale (HAMA-14), and the THINC-integrated tool (THINC-it) at baseline, post-treatment, and at one-week and four-week follow-up assessments. Exploratory resting-state functional magnetic resonance imaging (fMRI) was performed to investigate neuromodulatory effects.

**Results:**

Nine participants completed the study, corresponding to a completion rate of 75%, with a tolerability rate of 100%. No device-related adverse events were reported. Trends toward improvement were observed in depressive symptoms, anxiety symptoms, and subjective cognitive functioning. From baseline to post-treatment, HAMD-17 and HAMA-14 scores decreased by 52.0% and 56.7%, respectively, and these improvements were maintained at the four-week follow-up, with a 53.3% reduction in depression severity. Exploratory fMRI analyses demonstrated acute increases in functional connectivity between the right amygdala and sensorimotor cortical regions during the first stimulation session.

**Conclusions:**

A five-day course of right amygdala-targeted TIS was feasible, safe, and well tolerated in adolescents with depression. The observed improvements in depressive and anxiety symptoms, together with acute alterations in amygdala–cortical functional connectivity, provide hypothesis-generating signals that warrant further investigation in rigorously designed sham-controlled trials. However, given the open-label and uncontrolled nature of the study, these improvements cannot be causally attributed to the intervention.

**Clinical Trial Registration:**

The study has been registered on https://clinicaltrials.gov/ (registration number: No: NCT06452849; registration link: https://clinicaltrials.gov/study/NCT06452849).

## Main Points

1. A five‑day course of right amygdala‑targeting temporal interference stimulation (TIS) was found to be feasible and safe in adolescents with depression. All participants who initiated treatment completed the full course (n = 12), and no device‑related adverse events were reported.

2. In per‑protocol analysis (n = 9), depressive symptoms showed a 53.3% reduction at the four‑week follow‑up; intention‑to‑treat sensitivity analyses (n = 11) yielded a 43.6% reduction. Comparable reductions were observed for anxiety symptoms. These exploratory observations warrant further investigation.

3. Acute increases in amygdala‑cortical functional connectivity after the first TIS session were associated with subsequent changes in clinical scores at the four‑week follow‑up (Spearman’s ρ = –0.812). This preliminary observation provides a rationale for future studies.

## 1. Introduction

Depression is characterized by persistent sadness, anhedonia, and cognitive dysfunction [1], and it affects a large proportion of adolescents worldwide. According to the World Health Organization (2025), approximately 14.3% of adolescents (i.e., individuals aged 10 to 19 years) experience mental health conditions, one of the most frequent of them being depression [2]. The adolescent brain exhibits unique neurobiological vulnerability due to the ongoing maturation of prefrontal-limbic circuits and heightened stress reactivity, which predisposes adolescents to depression triggers ranging from academic pressure to social media exposure [3]. If left untreated, adolescent depression confers long-term risks, including educational underachievement, substance abuse, and suicidal behaviors [4].

Current first-line treatments for adolescent depression primarily involve pharmacotherapy and psychotherapy [5]. However, some adolescent patients experience treatment-refractory depression after sequential pharmacological treatments or psychotherapy, highlighting the need for novel therapeutic strategies. Neuromodulation techniques, such as repeated transcranial magnetic stimulation (rTMS) and deep brain stimulation (DBS), have shown promise for treating depression [6,7]. However, rTMS is constrained by its shallow penetration depth, preventing the direct modulation of key limbic structures, while DBS—though capable of precise subcortical targeting—requires invasive hardware implantation and is therefore less acceptable for adolescent populations.

Temporal interference stimulation (TIS) is a transformative neuromodulation approach that combines non-invasiveness with precise deep-brain targeting capabilities [8]. It employs two pairs of scalp electrodes to deliver high-frequency alternating currents with slight frequency differences (e.g., 2000 Hz and 2005 Hz). The intersection of these currents within the brain generates a low-frequency (e.g., 5 Hz) envelope capable of selectively modulating neuronal activity in deep structures without stimulating overlying cortical regions [8]. Growing evidence supports the therapeutic potential of TIS for neuropsychiatric diseases. Preclinical studies have demonstrated its efficacy in modulating addiction-related behaviors via nucleus accumbens (NAc) stimulation [9] and in enhancing motor recovery via primary motor cortex targeting [10,11]. In humans, TIS enhances motor skill learning via striatal modulation [12], improves episodic memory through hippocampal targeting [13], alleviates motor symptoms in Parkinson’s disease (PD) [14], and improves depressive symptoms and cognitive functions in patients with bipolar affective disorder experiencing depressive episodes by targeting the NAc [15]. A randomized controlled trial protocol has been established to investigate TIS targeting the subgenual anterior cingulate cortex in major depressive disorder [16].

The amygdala, a core hub of the limbic system, plays a central role in depression pathophysiology, exhibiting hyperactivity in major depressive disorder (MDD) and driving excessive negative emotional processing and impaired stress responses [17]. Structural neuroimaging studies consistently report amygdala enlargement in untreated MDD patients, particularly those with early-life stress exposure [18]. The present pilot feasibility study assessed the acceptability, tolerability, safety, and procedural feasibility of right amygdala-targeting TIS in adolescents with depression. Clinical outcomes and resting‑state functional magnetic resonance imaging (fMRI) data were evaluated as exploratory, hypothesis-generating objectives to inform the design of future controlled trials.

## 2. Materials and Methods

### 2.1 Participants

**Fig. 1.**
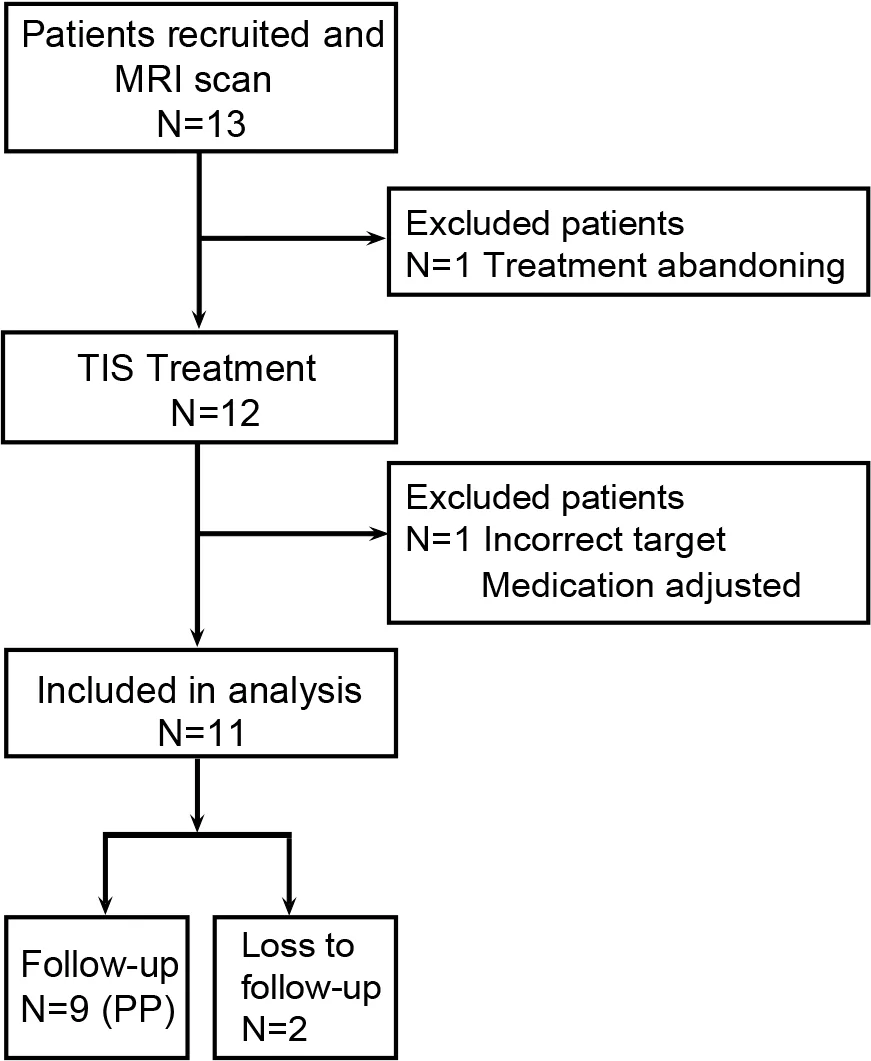
**The ﬂow chart of eligible patient inclusion**. MRI, magnetic resonance imaging; TIS, temporal interference stimulation; PP, per-protocol.

Adolescent depressive patients were recruited from the outpatient department for TIS treatment at Shanghai Pudong New Area Mental Health Center from June 2024 to January 2025. The inclusion criteria as follows: (i) Depressive disorder diagnosed based on the Diagnostic and Statistical Manual of Mental Disorders (DSM-5) criteria [19]; (ii) Age: 14–18 years; (iii) 17-item Hamilton Depression Rating Scale (HAMD-17) score ≥17; (iv) The antidepressant medication regimen remained unchanged from 30 days before signing the informed consent until the end of the trial; (v) In the judgment of the study physician, the subject or his/her agent understood the purpose and procedure of the study and consent comply with the study protocol requirements and sign the informed consent. The exclusion criteria as follows: (i) History of other psychiatric disorders, such as schizophrenia or bipolar disorder, which—according to the investigators—may have affected the outcome of the study; (ii) History of neurologic disorders, such as seizures; (iii) Metallic foreign bodies in the skull or metallic implants in the heart; (iv) Neurostimulation therapies such as electroconvulsive therapy, transcranial magnetic stimulation therapy or other physical therapy within the last three months; (v) Mental status assessed by the investigators as unstable and posing a considerable risk of suicide; (vi) Pregnancy or lactation; (vii) Participation in other clinical intervention trials; (viii) Other situations were considered inappropriate for the intervention in this study. The flow diagram of patient selection is shown in Fig. 1.

This single‑arm, open‑label pilot study primarily aimed to assess the feasibility (recruitment rate, adherence, completion rate), tolerability (adverse event profile), and safety of a five-day right amygdala–targeting TIS protocol in adolescents with depression. Secondary objectives, considered exploratory and hypothesis-generating, included the characterization of changes in depression and anxiety symptoms, cognitive function, and resting-state functional connectivity following TIS.

Of the 13 recruited adolescents, 12 initiated the TIS treatment protocol (one withdrew before starting treatment). All 12 participants who began treatment completed the five‑day course (100% treatment completion), and no one discontinued because of adverse events (100% tolerability). One participant was subsequently excluded from all outcome analyses because of a protocol violation (incorrect stimulation target and adjustment of antidepressant medication during the treatment period). Thus, the final analysis cohort consisted of 11 participants. Among these, nine completed all five treatment sessions and all follow‑up assessments (baseline, post‑treatment, one‑week, and four‑week) and were included in per‑protocol analyses. Two participants completed all five sessions but were not assessed post‑treatment after the baseline. All 12 participants who began treatment were included in feasibility and safety analyses.

### 2.2 Clinical Outcome Evaluation

Clinical outcomes were acquired before TIS treatment (baseline), after the last treatment (post-treatment), and at one- and four-week follow-ups. The severity of depressive symptoms was evaluated using the HAMD-17 [20] and the Montgomery-Åsberg Depression Rating Scale (MADRS) [21]. Patients were classified as responders if their HAMD-17 ratings decreased by at least 50% relative to the baseline. The severity of anxiety symptoms was evaluated using the 14-item Hamilton Anxiety Rating Scale (HAMA-14) [22]. The cognitive function was evaluated using the THINC-integrated tool (THINC-it) tool [23], which includes four objective cognitive tests (Spotter, Symbol Check, Code Breaker, and Trails) and a subjective cognitive questionnaire (Perceived Deficits Questionnaire for Depression-5-Item, PDQ-5-D).

### 2.3 MRI Image Acquisition and Preprocessing

The baseline assessment involved T1-weighted structural MRI. Scans were performed at the Department of Radiology, Shanghai Pudong New Area Mental Health Center, using a 3.0-T clinical scanner (United Imaging Healthcare, Shanghai, China). The patients lay supine with their heads snugly fixed by foam pads and were asked to keep still for as long as possible during scanning. T1-weighted images were obtained using a turbo field echo (TFE) sequence. The parameters are as follows: repetition time (TR) = 7.9 ms; echo time (TE) = 3.5 ms; inversion time = 900 ms; flip angle (FA) = 9°; field of view (FOV) = 240 × 240 mm^2^; matrix size = 320 × 256; voxel resolution = 1.00 × 1.00 × 1.00 mm^3^; total scan time = 4 minutes 41 seconds.

### 2.4 Electric Field Simulations

**Fig. 2.**
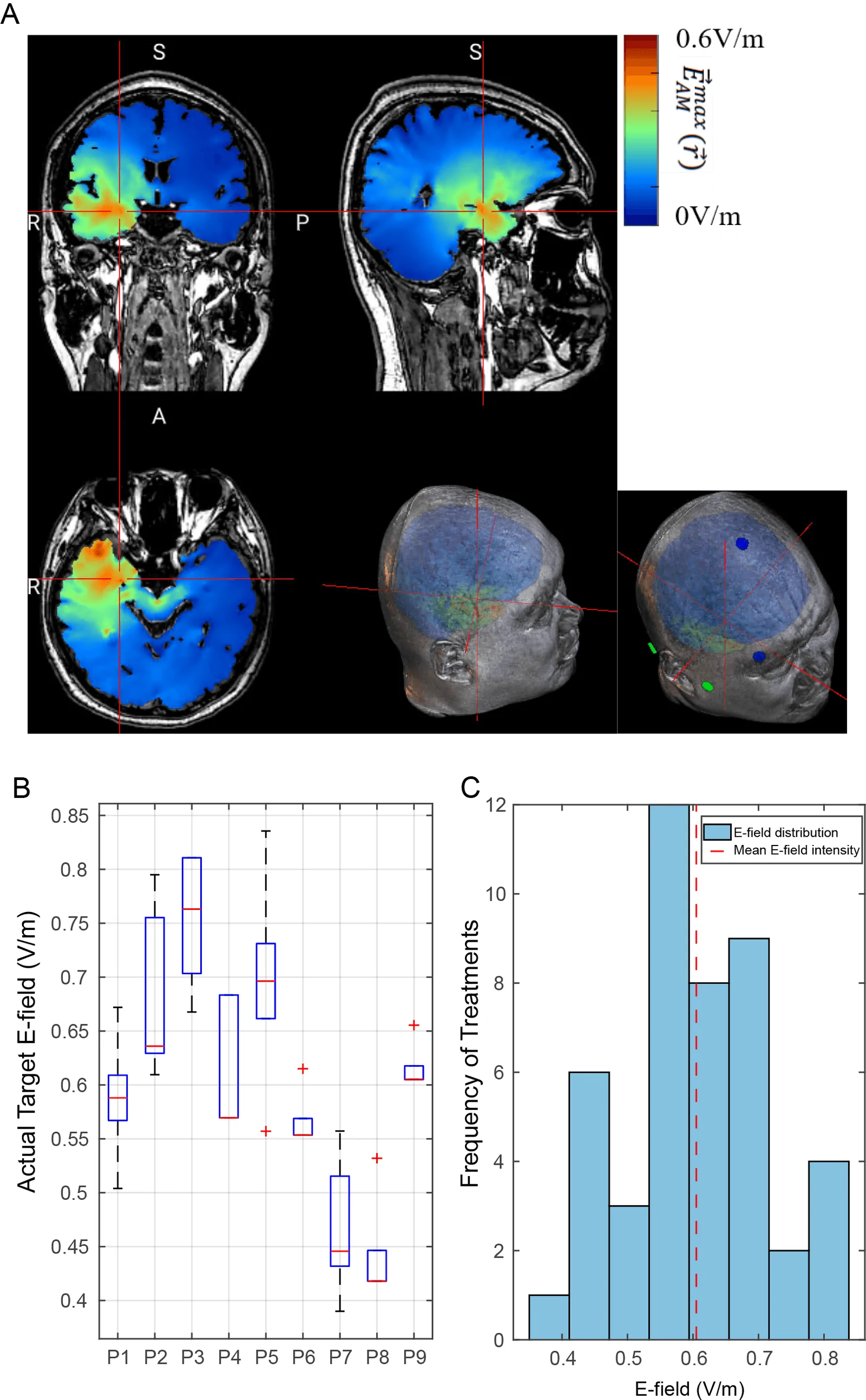
**Visualization and distribution of therapeutic electric field (E-field) for TIS**. (A) Electrode placement and electric field simulated by TI-pipeline. Bottom-right figures of each target depict the placement of two pairs of electrodes using the 10-10 system. Bottom-left, top-left, and top-right figures of each target illustrate the simulated strength of the electric field given the electrode placement and current proportion. Intersecting red crosshairs mark the target right amygdala for spatial registration; green dots and blue dots denote the scalp placement locations of the two pairs of stimulation electrodes. (B) Individual target E-field distribution across all sessions. Each box plot (P1 to P9) represents one participant, with the y-axis denoting the actual target E-field intensity (V/m) calculated for each treatment session. The boxes represent the interquartile range (IQR), red lines indicate the median, whiskers extend to 1.5× IQR, and red crosses denote outliers. (C) Pooled distribution of delivered E-field (N = 45 sessions). The histogram shows the frequency of treatments (y-axis) for each E-field intensity bin (x-axis). The red dashed line indicates the median E-field intensity for all 45 treatment sessions (nine patients × five sessions per patient).

The T1-weighted structural MRI images were segmented using SPM12 (Statistical Parametric Mapping; http://www.fil.ion.ucl.ac.uk/spm/). This procedure parcellated each participant’s head anatomy into six distinct tissue types and assigned corresponding conductivities: scalp (0.465 S/m), skull (0.01 S/m), cerebrospinal fluid (1.65 S/m), white matter (0.126 S/m), gray matter (0.276 S/m), and cavity (2.5 × 10^–14^ S/m). Following segmentation, the 10-10 international electroencephalography (EEG) electrode system was precisely coregistered to individual scalp surfaces. High-resolution tetrahedral finite element meshes were then generated from the segmented volumes using the Computational Geometry Algorithms Library (CGAL) [24]. After obtaining the meshes, we employed the finite element solver GetDP (https://github.com/hh-wu/getdp) [25] to compute the electric field intensity in the brains. This electric field intensity was calculated separately for each electrode pair configuration. Subsequently, the Temporal Interference (TI) electric field coupling principle [8] was applied to derive the TI electric field intensity. To identify stimulation targets, the Automated Anatomical Labeling (AAL3) atlas [26] was nonlinearly warped into individual MRI space using diffeomorphic registration. An exhaustive search method was used to identify the optimal electrode configurations for each participant, i.e., configurations where the electric field intensity was maximized at the target location (Fig. 2). Subsequent experiments were conducted based on these configurations. For each participant and session, the electric field intensity at the right amygdala was obtained from the finite element model using the corresponding current intensity as input. To account for the two-electrode-pair configuration, the simulated field intensity was divided by two and then multiplied by the maximum current intensity of the two channels. All simulations and post-processing steps were performed using the TI pipeline as described above. A threshold of 0.20 V/m, based on prior TIS optimization studies that used this value to quantify target coverage [27,28], was adopted to evaluate the proportion of sessions achieving a potentially effective field intensity at the amygdala.

### 2.5 TIS Procedures

TIS was delivered using the NervioX-1000 device (Suzhou Neurodome Medical Technology Co., Ltd. Suzhou, China). Personalized optimal TIS electrode locations and current intensities were calculated as described previously [12]. Participants received 20 min of right amygdala–targeting electrical stimulation per day for five consecutive days. Two pairs of electrodes delivered high-frequency currents with a carrier frequency of 2000 Hz for both channels, and the frequency difference between the two channels was set to 100 Hz (2000 Hz and 2100 Hz) to generate an interference envelope of 100 Hz at the target. A silver chloride powder electrode wire (outer diameter = 9.5 mm, inner diameter = 2.5 mm) was placed at the specific location by the operator for stimulation.

For each treatment session, current intensity was individually titrated. The intensity was increased in steps of 0.2 mA starting from 1.0 mA based on participant tolerability until the maximum comfortable level was reached. This titration procedure was repeated at the beginning of every session to adjust the current to the participant’s tolerance on each treatment day. The final current intensity delivered per session was recorded. The stimulator’s maximum output was 5 mA; the delivered current never reached this limit (range 1.4 to 3.4 mA).

### 2.6 Functional Connectivity Analysis

The collected BOLD-fMRI data were preprocessed using the SPM12 (https://www.fil.ion.ucl.ac.uk/spm/software/spm12/) and DPABI (http://rfmri.net/dpabi) toolkits. The specific procedures included: discarding the data of the first five TRs; temporal slice correction; head motion correction; spatial normalization (first registering the TIS image to the average functional image, then registering the T1 image to the Montreal Neurological Institute (MNI) standard template, and finally registering the functional image to the MNI standard space); smoothing the data with a Gaussian kernel of Full Width at Half Maximum (FWHM) 6 mm; removing the linear trend; regressing out 24 head motion parameters, white matter, and cerebrospinal fluid signals; filtering the data with a range of 0.01–0.1Hz; removing TRs with large head motion, using frame-wise displacement (FD) as the criterion, and deleting TRs with FD >0.2 mm for each subject. According to the AAL atlas, the right amygdala was selected as the seed point, and its whole-brain voxel-level functional connectivity was calculated to obtain the functional connectivity (FC) images of each subject based on the right amygdala. These images were used in subsequent statistical analysis. All imaging analyses were conducted as exploratory, hypothesis-generating objectives. For correlation analyses with clinical outcomes, we extracted individual-level functional connectivity changes (mean Fisher’s z-transformed values) from clusters showing substantial connectivity changes. As no clinical assessments were performed immediately after the first TIS session, we conducted exploratory prediction analyses examining whether early connectivity changes after the first session were associated with clinical outcomes at the four-week follow-up. Spearman’s rank correlation coefficients (ρ values) were calculated with 95% confidence intervals (CIs). Given the small sample size, these analyses are considered hypothesis-generating and require validation.

### 2.7 Missing Data Handling and Sensitivity Analyses

Given the nature of this pilot feasibility study, missing data were addressed using multiple approaches to evaluate the robustness of our findings. In per-protocol analysis: Primary clinical outcome analyses included only participants with complete data at all time points (n = 9). This approach provided estimates of clinical outcomes under ideal conditions. In the intention-to-treat (ITT) sensitivity analysis, all enrolled participants who received at least one TIS session and met eligibility criteria (n = 11) were included. For binary outcomes (responder status), missing follow-up data were imputed using non-responder imputation (worst-case scenario), with participants lost to follow-up classified as non-responders. In the case of continuous outcomes (HAMD-17, MADRS, HAMA-14 scores), baseline observation carried forward (BOCF) was applied for participants with missing post-treatment assessments, assuming no improvement from baseline. These conservative imputation strategies were chosen to avoid overestimation of clinical signals, consistent with the worst‑case principle and the exploratory nature of this pilot study.

### 2.8 Statistical Analysis

Given the pilot feasibility nature of the study and the very small sample size (n = 9 for per‑protocol analysis), inferential statistics (*p*‑values, *t*‑tests) were not used because they could produce misleadingly fragile results. Instead, we adopted a purely descriptive framework using 95% confidence intervals, which appropriately convey the precision and uncertainty of the estimates without implying causal inference. This approach follows the reporting standards recommended for single‑arm, small‑sample exploratory studies. Continuous variables (HAMD‑17, MADRS, HAMA‑14, THINC‑it scores) were reported as means with 95% CIs. Binary outcomes (responder rates) were reported as proportions with exact 95% CIs calculated using the Clopper‑Pearson method. The results of per‑protocol and ITT analyses were reported with denominators clearly specified.

For functional connectivity data, paired *t*‑tests were performed using SPM12 with Gaussian random field (GRF) correction (voxel‑*p* = 0.05, cluster‑*p* = 0.05). Spearman’s rank correlation was used to assess associations between these connectivity changes and clinical improvement (HAMD‑17 scores reduction at the four‑week follow‑up). GraphPad Prism 9 software (GraphPad Software, Inc., San Diego, CA, USA) was used to determine the descriptive statistics of clinical outcomes. Descriptive statistics for electric field simulation data were calculated using MATLAB 2022b (MathWorks, Natick, MA, USA).

## 3. Results

### 3.1 Demographics

The participants’ demographic and clinical characteristics are summarized in Table 1. The mean age was 16.7 ± 0.8 years (mean ± standard deviation [SD], range: 16 to 18 years), and the mean disease duration at treatment initiation was 16.0 ± 12.3 months (mean ± SD, range: 1 to 36 months).

**Table 1.** **Demographic information and clinical characteristics**.

Patient No.	Gender	Age	Education (year)	Depression duration (month)	Drug (mg/d)	Follow up
1	M	17	10	6	Sertraline 150 Buspirone 30 Quetiapine 25	5th TIS, 1 W, 4 W
2	F	18	11	24	Fluoxetine	5th TIS, 1 W, 4W
3	F	17	10	1	/	No
4	F	17	11	24	Fluoxetine Fluvoxamine	No
5	M	18	11	36	Sertraline 100 Sodium Valproate 500 Olanzapine 10	5th TIS, 1 W, 4 W
6	F	16	9	12	Venlafaxine 80 Quetiapine	5th TIS, 1 W, 4 W
7	M	16	10	2	Escitalopram 10 Alprazolam 0.4 Lorazepam 0.25	5th TIS, 1 W, 4 W
8	M	16	9	12	/	5th TIS, 1 W, 4 W
9	F	16	11	36	Escitalopram Quetiapine	5th TIS, 4 W
10	M	16	9	12	Sertraline Sodium Valproate	5th TIS, 4 W
11	M	17	11	11	/	5th TIS, 1 W, 4 W
Mean ± SD		16.7 ± 0.8	10.2 ± 0.9	16.0 ± 12.3		

W, week; SD, standard deviation; F, female; M, male.

### 3.2 Current Intensity Delivered

The current intensity was titrated individually for each session as described in Section 2.5. Across all 45 sessions, the median current intensity was 2.16 mA (interquartile range [IQR]: 1.60 to 2.80 mA) for channel 1 and 2.40 mA (IQR: 2.00 to 2.80 mA) for channel 2. Across the nine participants included in per‑protocol analysis, the mean current intensity per participant (averaged over five sessions) ranged from 1.47 to 3.16 mA for channel 1 and from 1.68 to 3.16 mA for channel 2. Descriptive statistics are summarized in Table 2. Individual session-by-session current intensity data for each participant are provided in **Supplementary Table 1**.

**Table 2.** Current intensity delivered across treatment sessions*.

Statistic		Channel 1 (mA)	Channel 2 (mA)	Ratio (ch1/ch2)
All sessions (n = 45)				
	Range	1.40 to 3.40	1.40 to 3.40	/
	Mean ± SD	2.17 ± 0.61	2.33 ± 0.50	/
	Median (IQR)	2.16 (1.60 to 2.80)	2.40 (2.00 to 2.80)	/
Per-participant average (n = 9)				
	Range	1.47 to 3.16	1.68 to 3.16	0.70 to 1.00
	Mean ± SD	2.17 ± 0.60	2.33 ± 0.47	0.92 ± 0.11
	Median (IQR)	2.20 (1.68 to 2.58)	2.32 (2.00 to 2.58)	1.0 (0.90 to 1.00)

*Data are based on 9 participants, each receiving 5 treatment sessions. For ratio statistics, only per-participant averages are reported, as the ratio was constant across sessions for each participant.

### 3.3 Electric Field Simulation Outcomes

Individualized electric field simulations were performed for each of the 45 treatment sessions (nine participants × five sessions) using the corresponding current intensity as input. After applying the post‑processing step described in Section 2.4, the median electric field intensity at the right amygdala across all sessions was determined as 0.61 V/m (interquartile range: 0.55 to 0.68 V/m; range: 0.39 to 0.84 V/m). Individual median field intensities ranged from 0.42 V/m (participant 8) to 0.76 V/m (participant 3), illustrating inter‑individual variability (Fig. 2B). The pooled frequency distribution (Fig. 2C) demonstrated that most treatment sessions clustered around 0.6 V/m, demonstrating the successful standardization of the TIS protocol at the group level. Detailed individual participant data are provided in **Supplementary Table 2**.

### 3.4 Clinical Outcomes

#### 3.4.1 Depression Symptoms (Exploratory Outcome)

**Fig. 3.**
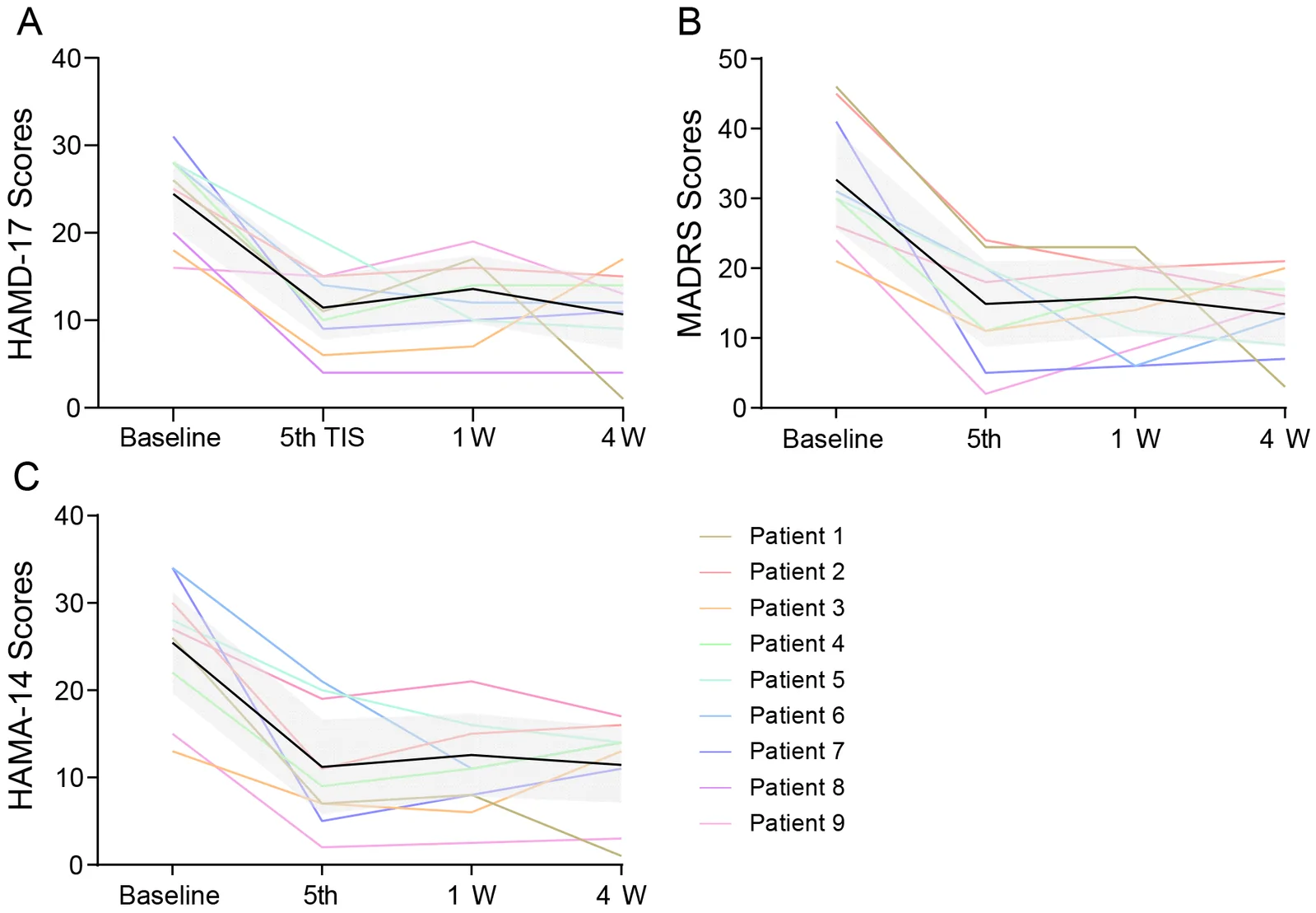
**Individual trajectories and group trends of clinical outcomes over time**. Individual trajectories of HAMD‑17 scores (A), MADRS scores (B), and HAMA-14 scores (C) for each participant (n = 9). The colored lines represent individual participants, the black line indicates the group means, and the light gray shaded area represents the 95% confidence interval. Scores decreased from baseline to post‑treatment and remained reduced at four‑week follow‑up. HAMA-14, 14-item Hamilton Anxiety Rating Scale; HAMD-17, 17-item Hamilton Depression Rating Scale; MADRS, Montgomery-Åsberg Depression Rating Scale.

The following findings are purely descriptive and exploratory and should not be interpreted as evidence of clinical efficacy. After the final treatment session, the HAMD-17 scores decreased by 52.0% (95% CI: 34.4% to 69.6%) compared with the baseline (24.4 ± 5.2 vs. 11.4 ± 4.8), and to a mean reduction of 13.0 points (95% CI: 8.4 to 17.6) (Fig. 3A). At the one-week follow-up, a 40.6% (95% CI: 14.1% to 67.1%) reduction was observed for the seven patients available for assessment (two patients were not followed up at one week) (24.1 ± 5.0 vs. 13.6 ± 4.2), with the mean reduction of 10.6 points (95% CI: 4.2 to 17.0). This reduction was sustained at the four-week follow-up, as evidenced by a 53.3% (95% CI: 31.3% to 75.3%) reduction compared with the baseline (24.4 ± 5.2 vs. 10.7 ± 5.2) and a mean reduction of 13.8 points (95% CI: 7.7 to 19.8) in per‑protocol analysis (n = 9) (Table 3). ITT analysis (n = 11) with BOCF yielded a mean reduction of 43.6% (95% CI: 21.1% to 66.1%) at the four‑week follow‑up.

**Table 3.** **Comparison of per-protocol and intention-to-treat analyses at 4-week follow-up**.

Outcome		Per-protocol (n = 9)	ITT (n = 11)*
HAMD-17			
	Mean reduction (%)	53.3% (31.3% to 75.3%)	43.6% (21.1% to 66.1%)
	Responder rate	66.7% (29.9% to 92.5%)	54.5% (23.4% to 83.3%)
MADRS			
	Mean reduction (%)	53.5% (33.0% to 74.1%)	43.8% (22.1% to 65.5%)
HAMA-14			
	Mean reduction (%)	52.5% (31.1% to 74.0%)	43.0% (21.0% to 65.0%)

ITT, intention-to-treat. Values are estimated (95% CI). *ITT analysis used baseline observation carried forward (BOCF) for continuous outcomes and non-responder imputation for binary outcomes.

In per‑protocol analysis (n = 9), the responder rate (≥50% reduction in HAMD‑17 score) was 77.8% (7/9; 95% CI: 40.0% to 97.2%) at post‑treatment, 57.1% (4/7; 95% CI: 18.4% to 90.1%) at the one‑week follow‑up, and 66.7% (6/9; 95% CI: 29.9% to 92.5%) at the four‑week follow‑up. ITT analysis (n = 11) using non‑responder imputation yielded responder rates of 63.6% (7/11; 95% CI: 30.8% to 89.1%), 36.4% (4/11; 95% CI: 10.9% to 69.2%), and 54.5% (6/11; 95% CI: 23.4% to 83.3%), respectively (Table 4). As expected for conservative imputation, ITT estimates were consistently lower than per‑protocol ones. The more pronounced reduction at the one‑week follow‑up (57.1% vs. 36.4%) reflects the inclusion of four participants with missing one‑week data (two who had fifth TIS and four‑week data but missed the one‑week assessment, and two with no follow-up assessments), all of whom were classified as non‑responders. At the primary endpoint, the ITT responder rate remained above 50% (54.5%), consistent with the direction of improvement observed in per‑protocol analysis.

**Table 4.** **Responder rates at each time point (per-protocol and ITT analyses)**.

Time point	Per-protocol (n = 9)	ITT (n = 11)*
5th TIS	77.8% (7/9; 40.0% to 97.2%)	63.6% (7/11; 30.8% to 89.1%)
1-week follow-up	57.1% (4/7; 18.4% to 90.1%)	36.4% (4/11; 10.9% to 69.2%)
4-week follow-up	66.7% (6/9; 29.9% to 92.5%)	54.5% (6/11; 23.4% to 83.3%)

Values are proportion (95% CI). 95% confidence intervals were calculated using the Clopper-Pearson exact method. *ITT analysis used non-responder imputation for missing follow-up data.

Consistent improvements were observed in terms of the MADRS scores (Fig. 3B). At the four‑week follow‑up, the mean score reduction was 19.2 points (95% CI: 9.2 to 29.3) or 53.5% (95% CI: 33.0% to 74.1%) in per‑protocol analysis (n = 9), while ITT analysis revealed a decrease of 15.7 points (95% CI: 6.3 to 25.2) or 43.8% (95% CI: 22.1% to 65.5%) (Table 3).

#### 3.4.2 Anxiety Symptoms (Exploratory Outcome)

HAMA-14 scores decreased by 56.7% (95% CI: 39.5% to 73.9%) after the final TIS session (25.4 ± 7.5 vs. 11.2 ± 7.0), with the mean reduction equaling 14.2 points (95% CI: 8.7 to 19.7) (Fig. 3C). At the four‑week follow‑up (25.4 ± 7.5 vs. 11.4 ± 5.6), the mean reduction was 14.0 points (95% CI: 8.0 to 20.0) and 52.5% (95% CI: 31.1% to 74.0%). ITT analysis (n = 11) with BOCF yielded a mean reduction of 43.0% (95% CI: 21.0% to 65.0%) at the four‑week follow‑up (Table 3).

#### 3.4.3 Cognitive Function (Exploratory Outcome)

Cognitive assessments revealed preliminary improvement following TIS. All THINC‑it scores (subtest and total scores) were reported as standardized scores (z‑scores) according to the official scoring algorithm, with higher scores indicating a better cognitive function. Changes from baseline to post‑treatment (fifth TIS) with 95% CIs are summarized in Table 5. The THINC‑it total scores showed improvement, increasing by 2.25 points on average (95% CI: 0.47 to 4.02). All objective subtests (Spotter, Symbol Check, Codebreaker, and Trails) also demonstrated numerical improvements, with mean changes ranging from 0.18 (Codebreaker) to 0.66 (Trails) points; however, the 95% CIs for these subtests crossed zero (Table 5), indicating that the improvements were modest and uncertain. The subjective cognitive measure (PDQ‑5‑D) also improved, with the mean increase of 0.75 points (95% CI: 0.15 to 1.35), suggesting a trend toward a better self‑perceived cognitive function.

**Table 5.** **The changed THINC-it score**.

Item	Baseline Mean ± SD	5th TIS Mean ± SD	Mean change (95% CI)
Spotter	–0.16 ± 0.83	0.16 ± 1.17	0.32 (–0.33 to 0.98)
Symbol Check	–0.17 ± 0.93	0.17 ± 0.29	0.33 (–0.26 to 0.93)
Codebreaker	–0.09 ± 0.98	0.09 ± 1.07	0.18 (–0.41 to 0.78)
Trails	–0.33 ± 1.30	0.33 ± 0.44	0.66 (–0.32 to 1.64)
PDQ-5-D	–0.37 ± 0.88	0.37 ± 1.02	0.75 (0.15 to 1.35)
THINC-it total score	–1.12 ± 3.41	1.12 ± 2.64	2.25 (0.47 to 4.02)

PDQ-5-D, Perceived Deficits Questionnaire for Depression-5-item; THINC-it, THINC-integrated tool.

### 3.5 Functional Connections (Exploratory Analysis)

**Fig. 4.**
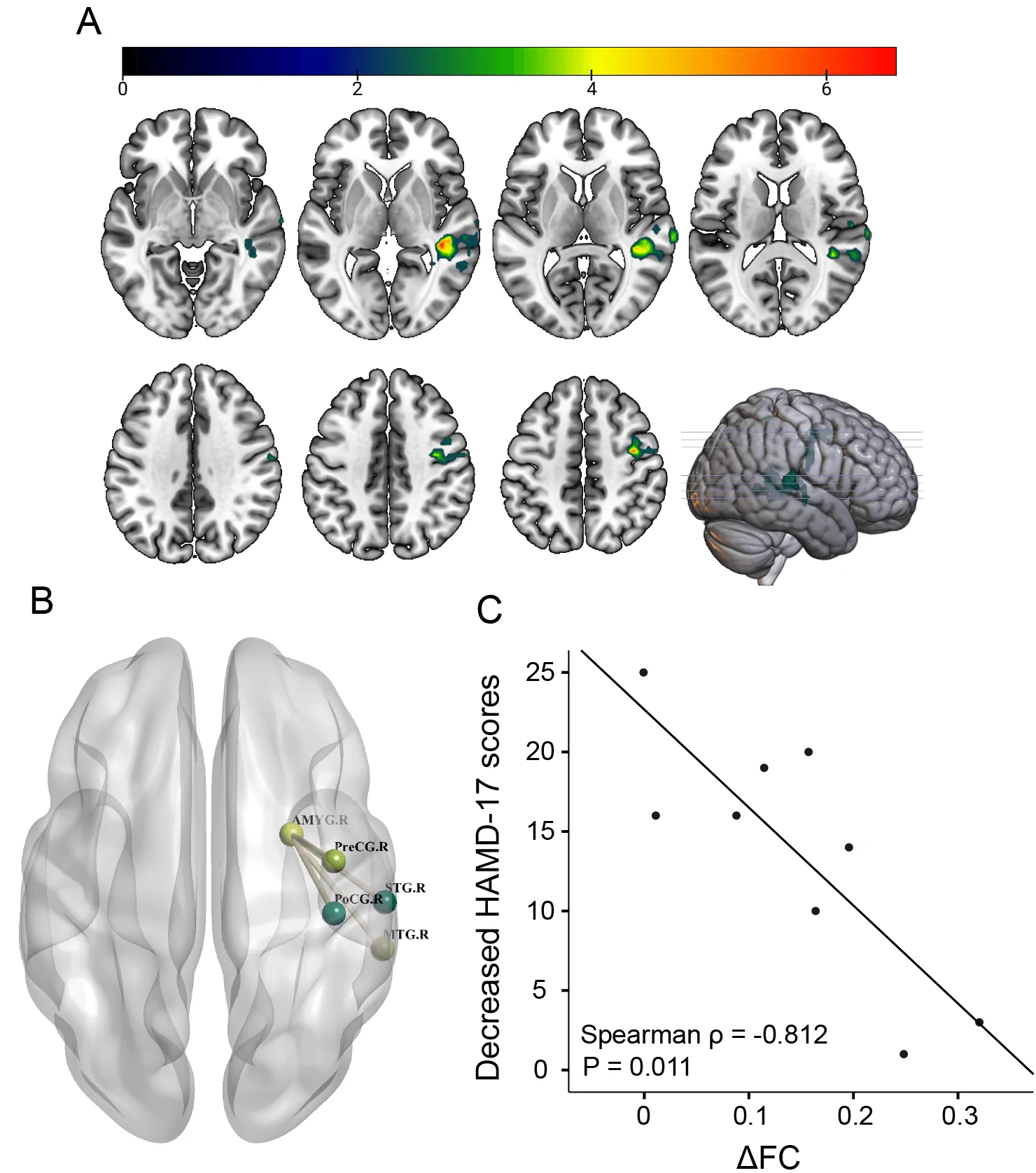
**Functional connectivity with the right amygdala as the seed point**. (A) The T-value graph of FC comparison. First session, right amygdala TIS increased the FC between the right amygdala and right superior temporal gyrus, right precentral gyrus, right middle temporal gyrus, and right postcentral gyrus compared to the baseline. The T value, ranging from 0 to 6, indicates an increase in the difference in FC. (B) Single-view functional connection diagram. (C) Exploratory prediction analysis, Spearman correlation between acute functional connectivity changes (right precentral gyrus-amygdala) and HAMD-17 scores reduction at four-week follow-up (n = 9). A strong negative correlation was observed (ρ = –0.812, exact *p* = 0.011). AMYG.R, right amygdala; FC, functional connectivity; PreCG.R, right precentral gyrus; PoCG.R, right postcentral gyrus; MTG.R, right middle temporal gyrus. (voxel-*p* = 0.05, cluster-*p* = 0.05, Gaussian random field (GRF) corrected).

As an exploratory, hypothesis-generating objective, we examined changes in resting-state functional connectivity following TIS using seed-based analysis with the right amygdala as the seed region. Exploratory analyses revealed that compared with baseline, the first TIS session was associated with increased functional connectivity between the right amygdala and a cluster of regions, including the right superior temporal gyrus, precentral gyrus, middle temporal gyrus, and postcentral gyrus (GRF-corrected) (Fig. 4A,B), whereas no such changes were observed during the fifth TIS session.

As clinical assessments were not performed immediately after the first TIS session, we conducted exploratory prediction analyses to examine whether early connectivity changes were associated with clinical outcomes at the four‑week follow‑up. Among the four regions, the right precentral gyrus (PreCG_R)-amygdala circuit showed a notable negative correlation with the reduction in HAMD‑17 scores at the four‑week follow‑up (Spearman’s ρ = –0.812; Fig. 4C). Correlations for the other three regions were weak and non‑significant. Given the small sample size, this finding should be interpreted as hypothesis‑generating and requires validation in larger cohorts.

### 3.6 Adverse Events

The TIS treatment was well-tolerated, with no serious adverse events reported during the study or follow-up period. Of the nine participants, seven reported a total of 31 adverse events throughout the five treatment sessions. Among these, 19 events in five participants were considered stimulus-related. Details about individual adverse events are provided in **Supplementary Table 3**.

## 4. Discussion

Adolescent depression is an important mental health condition that requires early intervention and innovative treatment. This pilot feasibility study demonstrates that a five-day regimen of right amygdala-targeting TIS is safe, well-tolerated (100% tolerability among completers), and procedurally feasible in adolescents with depression. To our knowledge, this is the first investigation to apply TIS to adolescent depression, an area where non-invasive neuromodulation options are limited. As exploratory, hypothesis-generating objectives, we characterized changes in clinical symptoms and functional connectivity, observing notable reductions in HAMD-17 scores (53.3% at the four-week follow-up). However, given the open-label, uncontrolled design and small sample size, these clinical improvements cannot be causally attributed to the intervention and may reflect placebo effects, natural history, or regression to the mean. Nonetheless, these conservative imputation strategies provide lower‑bound estimates of treatment effects; the fact that the primary endpoint (four-week follow‑up) still showed a >50% responder rate under the worst‑case assumption supports the robustness of the exploratory findings.

Of the 13 recruited adolescents, 12 completed the full TIS course (92% acceptance). One participant was excluded from outcome analyses because of a protocol violation (incorrect stimulation target), leaving 11 participants for ITT analysis, of whom nine completed all follow‑up assessments and were included in per‑protocol analysis. Two participants were lost to follow-up. The requirement for daily hospital visits over a short period, coupled with the need for eight total visits throughout the study, may pose substantial burdens for school-aged adolescents and their families. To improve feasibility, future studies may consider implementing accelerated TIS protocols with multiple daily sessions to reduce overall treatment duration, as has been successfully demonstrated for TMS [29]. Furthermore, the development of portable, home-based TIS devices could fundamentally overcome accessibility barriers.

Individualized electric field simulations confirmed that the right amygdala was exposed to the TIS field in all 45 treatment sessions, with a median field intensity of 0.61 V/m (IQR: 0.55–0.68 V/m). All sessions achieved field intensities above the 0.20 V/m reference threshold used to quantify target coverage in prior TIS modeling studies [27,28]. These values are comparable with the field strengths predicted for other deep-brain targets (e.g., pallidum, hippocampus, striatum) stimulated using similar electrode configurations [12,28], indicating that our stimulation parameters were capable of delivering an electric field of a magnitude similar to those reported in previous computational work. It is important to note, however, that the 0.20 V/m threshold is a modeling reference value for standardizing target coverage assessment, not a proven minimum effective field intensity. Moreover, the observed inter‑individual variability (individual median field intensities ranging from 0.42 to 0.76 V/m) may contribute to heterogeneity in clinical response. Participants with lower target field strengths might have received relatively lower exposure, which could affect treatment outcomes. Future studies with larger sample sizes should directly examine the correlation between individualized electric field intensity at the amygdala and clinical response, which would help establish a dose‑response relationship and guide personalized stimulation protocols. Such studies should also explore individualized dosing strategies, for example, using subject‑specific head models or adaptive titration protocols to ensure consistent target engagement.

A key exploratory observation was the acute increase in functional connectivity between the right amygdala and a distributed cortical network (including the right superior temporal gyrus, precentral gyrus, middle temporal gyrus, and postcentral gyrus) after the first TIS (but not the fifth) session. Notably, the increased connectivity between the right amygdala and precentral gyrus during the first session correlated with subsequent clinical improvement (ρ = –0.812). Given the small sample size and the lack of concurrent clinical assessments at the time of fMRI acquisition, these findings are hypothesis‑generating. Future studies with larger samples, sham‑controlled designs, and multi‑timepoint imaging‑clinical assessments are needed to further investigate the relationship between connectivity changes and clinical response to TIS.

Previous studies have examined the effects of TMS and tDCS on adolescent depression [30,31]. Unlike these techniques, which have limited penetration depth, TIS can target deep structures such as the amygdala—a feature that may contribute to its favorable tolerability profile in this preliminary investigation. The occurrence of depression involves multiple brain regions, including subcortical regions. TIS can stimulate deep regions and thus help increase the diversity of available therapeutic targets. The adolescent brain, characterized by heightened neuroplasticity, may be particularly responsive to such neuromodulation. The rapid functional connectivity changes and early cognitive improvement observed herein are consistent with models of targeted network modulation. For instance, selective serotonin reuptake inhibitors (SSRIs) rapidly reduce amygdala hyperactivity, correlating with early symptom improvement [32], and cognitive-behavioral therapy (CBT) enhances ventromedial prefrontal cortex (vmPFC)-amygdala inhibitory connectivity, promoting emotion regulation [33]. Patients with treatment-refractory depression (TRD) have shown decreased right-amygdala responsivity, and the long-term DBS of the ventral anterior limb of the internal capsule normalized this responsivity [34]. Future studies should integrate multimodal neuroimaging to further elucidate the causal mechanisms of TIS.

Regarding safety, the most common side effects were mild to moderate sleepiness and headache, which were transient and self-limiting. No serious adverse events were observed, which confirms the safety of our TIS protocol for adolescents.

### Limitations

This pilot study had several limitations. First, the small sample size (n = 9 for per‑protocol analysis and n = 11 for ITT) and open-label design mean that the findings should be interpreted with caution. Larger randomized sham-controlled trials are needed to validate our findings. Second, the heterogeneity of concomitant pharmacological treatments across participants (Table 1) represents an additional interpretive limitation. The potential interaction between medication and TIS could not be disentangled in this small sample. Future controlled trials with larger, more homogeneous cohorts are needed to isolate the specific effects of TIS. Third, the lack of a long-term follow-up beyond four weeks limits the understanding of the durability of the observed clinical signals. Fourth, the notable inter-individual variability in differences in clinical response (e.g., one participant showed almost no improvement throughout the follow-up period) and in simulated electric field strength at the target observed herein suggests that individual differences in amygdala morphology or baseline functional activity may influence treatment response. This hypothesis could be directly tested in future studies using baseline structural and functional neuroimaging. Fifth, although ITT sensitivity analyses using conservative imputation (non‑responder imputation for binary outcomes and BOCF for continuous outcomes) yielded estimates directionally consistent with per‑protocol findings, the greater reduction in the responder rate at the one‑week follow‑up reflects the application of conservative imputation to a larger proportion of missing data at that time point. These analyses suggest that dropouts did not fundamentally alter the overall pattern of results, although the small sample size limits the precision of these estimates.

## 5. Conclusions

The five-day course of right amygdala-targeted TIS was found to be safe and well-tolerated in adolescents with depression. As exploratory observations, preliminary improvements in depression and anxiety symptoms were noted alongside acute changes in amygdala‑cortical functional connectivity. Given the open‑label design of this study and small sample size, these findings cannot be attributed to the intervention with certainty. Future studies with larger samples, active control conditions, and mechanistic neuroimaging protocols are needed to determine whether TIS provides clinical benefit in adolescents.

## Data Availability

The data used and analyzed during the current study are available from the corresponding author upon reasonable request.
