# Supplementary material for: Temporal Interference Stimulation Targeting the Right Amygdala in Adolescents With Depression: A Pilot Feasibility Study
Source: Alpha Psychiatry. 2026 Jul 15;27(4):50313. doi: 10.31083/AP50313 (PMC13539988; doi:10.31083/AP50313)
Supplement: Supplementary file 1 [file 2757-8038-27-4-50313-s1.zip › Supplementary Material.docx]

**Temporal Interference Stimulation Targeting the Right Amygdala in Adolescents with Depression: A Pilot Feasibility Study**

**Supplementary Table 1. Individual current intensities per session**

| Participant | Session | Channel 1 (mA) | Channel 2 (mA) | Ratio (ch1/ch2) |
| --- | --- | --- | --- | --- |
| 1 | 1 | 2.4 | 2.4 | 1 |
|  | 2 | 2.8 | 2.8 | 1 |
|  | 3 | 2.8 | 2.8 | 1 |
|  | 4 | 2.8 | 2.8 | 1 |
|  | 5 | 3.2 | 3.2 | 1 |
| 2 | 1 | 2.3 | 2.3 | 1 |
|  | 2 | 2.4 | 2.4 | 1 |
|  | 3 | 2.4 | 2.4 | 1 |
|  | 4 | 2.4 | 2.4 | 1 |
|  | 5 | 3 | 3 | 1 |
| 3 | 1 | 2.8 | 2.8 | 1 |
|  | 2 | 3 | 3 | 1 |
|  | 3 | 3.2 | 3.2 | 1 |
|  | 4 | 3.4 | 3.4 | 1 |
|  | 5 | 3.4 | 3.4 | 1 |
| 4 | 1 | 1.4 | 2 | 0.7 |
|  | 2 | 1.4 | 2 | 0.7 |
|  | 3 | 1.4 | 2 | 0.7 |
|  | 4 | 1.68 | 2.4 | 0.7 |
|  | 5 | 1.68 | 2.4 | 0.7 |
| 5 | 1 | 1.44 | 1.6 | 0.9 |
|  | 2 | 1.8 | 2 | 0.9 |
|  | 3 | 1.8 | 2 | 0.9 |
|  | 4 | 1.8 | 2 | 0.9 |
|  | 5 | 2.16 | 2.4 | 0.9 |
| 6 | 1 | 1.44 | 1.8 | 0.8 |
|  | 2 | 1.44 | 1.8 | 0.8 |
|  | 3 | 1.44 | 1.8 | 0.8 |
|  | 4 | 1.44 | 1.8 | 0.8 |
|  | 5 | 1.6 | 2 | 0.8 |
| 7 | 1 | 1.4 | 1.4 | 1 |
|  | 2 | 1.6 | 1.6 | 1 |
|  | 3 | 1.6 | 1.6 | 1 |
|  | 4 | 1.8 | 1.8 | 1 |
|  | 5 | 2 | 2 | 1 |
| 8 | 1 | 2.2 | 2.2 | 1 |
|  | 2 | 2.2 | 2.2 | 1 |
|  | 3 | 2.2 | 2.2 | 1 |
|  | 4 | 2.2 | 2.2 | 1 |
|  | 5 | 2.8 | 2.8 | 1 |
| 9 | 1 | 2.16 | 2.4 | 0.9 |
|  | 2 | 2.16 | 2.4 | 0.9 |
|  | 3 | 2.16 | 2.4 | 0.9 |
|  | 4 | 2.16 | 2.4 | 0.9 |
|  | 5 | 2.34 | 2.6 | 0.9 |

**Supplementary Table 2. Individual electric field intensities for each participant across five treatment sessions**

| Participant | Session 1 (V/m) | Session 2 (V/m) | Session 3 (V/m) | Session 4 (V/m) | Session 5 (V/m) | Individual median (V/m) |
| --- | --- | --- | --- | --- | --- | --- |
| 1 | 0.50 | 0.59 | 0.59 | 0.59 | 0.67 | 0.59 |
| 2 | 0.61 | 0.64 | 0.64 | 0.74 | 0.80 | 0.64 |
| 3 | 0.67 | 0.72 | 0.76 | 0.81 | 0.81 | 0.76 |
| 4 | 0.57 | 0.57 | 0.57 | 0.68 | 0.68 | 0.57 |
| 5 | 0.56 | 0.70 | 0.70 | 0.70 | 0.83 | 0.70 |
| 6 | 0.55 | 0.55 | 0.55 | 0.55 | 0.62 | 0.55 |
| 7 | 0.39 | 0.45 | 0.45 | 0.50 | 0.56 | 0.45 |
| 8 | 0.42 | 0.42 | 0.42 | 0.42 | 0.53 | 0.42 |
| 9 | 0.61 | 0.61 | 0.61 | 0.61 | 0.61 | 0.61 |

**Supplemental Table 3. Detailed Adverse Events by Study Phase**

| Treatment No. | Patient No. | Brief description/Subjective | Serious level | Anticipated consent | Study relatedness |
| --- | --- | --- | --- | --- | --- |
| 1^st^ TIS | 1 | Sleepiness | Mild | Yes | No |
|  | 2,7 | Sleepiness | Moderate | Yes | No |
|  | 2 | Dizziness | Mild | Yes | No |
|  | 4 | Sleepiness | Mild | Yes | Yes |
|  | 5 | Headache | Mild | Yes | Yes |
|  | 5 | Pruritus | Mild | Yes | Yes |
|  | 9 | Sleepiness | Moderate | Yes | Yes |
| 2^nd^ TIS | 1 | Sleepiness | Mild | Yes | No |
|  | 4 | Nausea/gastrointestinal discomfort | Mild | Yes | No |
|  | 5 | Headache | Mild | Yes | Yes |
|  | 5 | Nausea/gastrointestinal discomfort | Mild | Yes | No |
|  | 5 | Sleepiness | Mild | Yes | No |
|  | 8 | Tingle | Mild | Yes | Yes |
|  | 9 | Unusual sensation on the scalp | Moderate | Yes | Yes |
| 3^rd^ TIS | 1 | Sleepiness | Moderate | Yes | No |
|  | 2 | Unusual sensation on the scalp | Mild | Yes | Yes |
|  | 2 | Pruritus | Mild | Yes | Yes |
|  | 4 | Sleepiness | Mild | Yes | No |
|  | 5 | Headache | Mild | Yes | Yes |
|  | 8 | Tingle | Mild | Yes | Yes |
|  | 9 | Tingle | Moderate | Yes | Yes |
| 4^th^ TIS | 2 | Neck pain | Mild | Yes | No |
|  | 4 | Sleepiness | Mild | Yes | No |
|  | 5 | Headache | Mild | Yes | Yes |
|  | 9 | Tingle | Heavy | Yes | Yes |
|  | 9 | Anxiety/worry/nervousness | Mild | Yes | Yes |
| 5^th^ TIS | 2 | Unusual sensation on the scalp | Mild | Yes | Yes |
|  | 4 | Sleepiness | Mild | Yes | No |
|  | 5 | Headache | Mild | Yes | Yes |
|  | 9 | Tingle | Moderate | Yes | Yes |
|  | 9 | Pruritus | Moderate | Yes | Yes |
